# The effect of core self-evaluations on career adaptability: The mediating role of protean career attitudes and the moderating role of meritocratic beliefs

**DOI:** 10.3389/fpsyg.2022.1000615

**Published:** 2022-11-24

**Authors:** Bin Du, Xuan Yu, Nan Luo, Xuhong Liu

**Affiliations:** ^1^Recruitment and Employment Office, Sichuan Agricultural University, Chengdu, China; ^2^School of Economics and Management, Southwest Petroleum University, Chengdu, China; ^3^Business School, Sichuan University, Chengdu, China; ^4^Department of Police Management, Sichuan Police College, Luzhou, China

**Keywords:** core self-evaluations, career adaptability, protean career attitudes, meritocratic beliefs, college students, core

## Abstract

Based on the career construction model of adaptation, this study explores the impact of core self-evaluations on career adaptability, with the mediating role of protean career attitudes and moderating role of meritocratic beliefs. The results of the questionnaire survey on 1000 Chinese college students show that: (1) core self-evaluations positively predicted college students’ career adaptability; (2) protean career attitudes mediated the relationship between core self-evaluations and career adaptability; (3) meritocratic beliefs not only moderated the effect protean career attitudes have on career adaptability but also moderated the indirect influence of core self-evaluations on career adaptability through protean career attitudes. These results extend the existing antecedent studies on career adaptability and demonstrate the importance of combining self-mobility beliefs (protean career attitudes) with social mobility beliefs (meritocratic beliefs) in the process of core self-evaluations affecting career adaptability. In conclusion, we hope to further develop the theory of career construction and provide more suggestions for college consultants and students.

## Introduction

In the current era of globalization, young people are facing an unstable labor market and an unpredictable career environment (Zacher, 2014; Chui et al., 2020). Career adaptability, as a psychological resource for coping with current and anticipated tasks, transitions, and traumas in their occupational roles (Savickas and Porfeli, 2012), which consists of four dimensions: career concern (thinking and preparing for future career orientation), career control (making deliberate decisions and taking conscientious actions), career curiosity (the ability to pursue career exploration), and career confidence (the positive belief into one’s problem-solving skills), can help individuals to adapt to new work demands, diverse groups, and different environments in the rapidly changing career landscape (Savickas et al., 2009). Therefore, it is necessary to pay attention to the career adaptability of college students to help them cope with the challenges of uncertain careers. Specifically, our study will focus on the overall career adaptability of college students, and the validity of career adaptability as a whole construct has been confirmed in previous studies (Cai et al., 2015; Guan et al., 2015), thus, career adaptability will not be discussed in different dimensions in the following argument.

Career adaptability is the core concept of career construction theory, which holds that the individual adaptation process includes four stages: adaptive readiness (i.e., adaptivity), adaptability, adapting, and adaptation (Savickas and Porfeli, 2012), among them, adaptive preparation usually includes cognitive ability and personality traits (Rudolph et al., 2017a,b). In the current research, there is no consensus on the impact of cognitive ability on career adaptability. However, personality traits are generally considered to have a significant relationship with career adaptability (Savickas, 2005; Savickas, 2013). Previous studies have verified that the Big Five personality traits (Li et al., 2015), proactive personality, future orientation (Rudolph et al., 2017a,b) and time focus (Pouyaud et al., 2012) all have significant effects on career adaptability. With the development of career construction theory, core self-evaluations, as a positive self-trait, have attracted extensive attention from researchers on its influence on attitude, motivation, and career development (Judge and Kammeyer-Mueller, 2011). Core self-evaluations represent the fundamental appraisals individuals make about their self-worth and capabilities, conceptualized as a higher order construct composed of broad and evaluative traits (e.g., self-esteem and generalized self-efficacy). In the framework of career construction theory, core self-evaluations are regarded as an individual’s adaptive readiness and have a significant positive impact on adaptability resources (Neureiter and Traut-Mattausch, 2017).

Although the positive impact of core self-evaluations on career adaptability has been confirmed in relevant studies (Zacher, 2014; Rudolph et al., 2017a,b), it is still unknown how this effect is generated. In other words, the mediating mechanism between core self-evaluations and career adaptability is still unclear and needs to be explored. The last two decades of career research have shown that contemporary careers require approaches that are adaptive, proactive and self-managed in order to cope with the increased uncertainty, mobility and boundarylessness of work (Gubler et al., 2014). In other words, it is individuals rather than organizations that dominate career development. People no longer choose employers or jobs based on the needs of the organization but on their own career development goals (Briscoe et al., 2006). The transition of individuals from self-organized based on social career norms to self-extend based on career growth planning reflects the process of individual career construction (Savickas, 2005; Savickas and Porfeli, 2012; Savickas, 2013), and this process also reflects the implication of protean career attitudes. Protean career attitudes are attitudes of freedom, autonomy, and making choices based on one’s own values, which can be divided into two dimensions of self-orientation and value-orientation (Briscoe and Hall, 2006). Researchers believe that it can help college students improve their insecurity in an uncertain career environment so as to better navigate new career prospects (Higgins et al., 2010). Moreover, according to the distal-proximal framework of motivation (Kanfer, 1990), core self-evaluations as distal stable traits may affect college students’ career adaptability through their proximal motivation. Since previous studies have shown that core self-evaluations can influence protean career attitudes (Rodrigues et al., 2019), and the protean career attitudes just represent the proximal motivation of individuals to career self-organization (Direnzo et al., 2015), which makes students more easily adapt to the changeable environment (Chan et al., 2015). Therefore, we think that protean career attitudes may explain how students’ core self-evaluations affect their career adaptability in current career development.

Furthermore, the protean career attitudes not only represents the individual’s self-control over the career, but also makes it easier for individuals to move between different occupations or organizations to adapt to the dynamic labor market (Gubler et al., 2014; Redondo et al., 2021), however, individuals’ perceived control over the external world and perceived mobility may affect individuals with protean career attitudes to translate their intention to pursue career development into actual behaviors. To some extent, meritocratic beliefs, as an individual’s cognition of the principle of distribution of social results, represent people’s perception of the controllability of the external world and the possibility of social mobility (Shane and Heckhausen, 2017). As individuals who hold such beliefs believe that they can move freely in society by their own efforts or abilities (Hu et al., 2020), their protean career attitudes may have a more significant effect on career adaptability. On the contrary, once individuals with protean career attitudes believe that the possibility of social mobility is reduced and the environment is out of their control, they are also difficult to adapt to the uncertain environment, because they believe that there are external constraints in finding opportunities (Smith and Stone, 1989; Shane and Heckhausen, 2015). Based on this understanding, we speculated that meritocratic beliefs might play a moderating role in the relationship between core self-evaluation and protean career attitudes.

Therefore, this study intends to clarify the influence of college students’ core self-evaluations on career adaptability with protean career attitudes as the mediator and meritocratic beliefs as the moderator, hoping to provide some help for career consultation in colleges and universities. In the face of the challenges brought by the uncertain labor market, career counselors need to guide students to establish meritocratic beliefs, and tailor career adaptability training programs for stjudents with different core self-evaluations levels by cultivating students’ protean career attitudes.

### Core self-evaluations and career adaptability

Career adaptability is defined as the psychological resources that help regulate an individual’s goal-pursuing process during various career transitions, including control, control, curiosity, and confidence (Savickas, 1997). As a powerful predictor of career adaptability, core self-evaluations refer to people’s comprehensive evaluation of their self-value and abilities (Judge et al., 1998), which includes four dimensions: self-esteem, generalized self-efficacy, locus of control, and (low) neuroticism or emotional stability (Judge et al., 2002).

Previous studies have pointed out that individuals with high core self-evaluations show more initiative, persistence and high commitment to goals when pursuing goals (Gagné and Deci, 2005), and can effectively self-regulate according to the environment in order to achieve goals (Chang et al., 2011). Moreover, individuals with a higher level of core self-evaluations have a lower level of stress (Kammeyer-Mueller et al., 2009). To some extent, they think that there is almost no threat in the environment or regard threats as opportunities so that they can adopt coping strategies more effectively to adapt to changes in the environment (Judge et al., 1999). Therefore, high level of core self-evaluations can bring college students stronger career adaptability (Öncel, 2014; Hirschi et al., 2015).

According to career construction theory (Savickas, 2005; Savickas and Porfeli, 2012), those who are willing or ready (i.e., adaptivity) to change and have the psychosocial resources (i.e., adaptability) to do so are better able to make adaptive responses/behaviors to changing conditions thereby achieving positive adaptive outcomes (Savickas, 2013). Existing studies believe that core self-evaluations can be considered as a kind of adaptive readiness (Neureiter and Traut-Mattausch, 2017; Rudolph et al., 2017a,b). College students with a high level of core self-evaluations can independently guide their behaviors and adjust their performance in a complex and ambiguous environment (Judge and Kammeyer-Mueller, 2011), thus showing a higher level of career adaptability. Thus, we proposed the following:

*Hypothesis 1*: core self-evaluations will be positively associated with career adaptability.

### Mediating role of protean career attitudes

Protean career attitudes include two dimensions: self-directed, which expresses the degree to which a person controls and manages his/her own career (Mirvis and Hall, 1994), and value-driven, which closely combines career decisions with individuals’ values, rather than objective rewards or the values of others (Briscoe et al., 2006).

Core self-evaluations, as meta-traits that captures positive self-concept (Judge et al., 2003), can help individuals acquire sufficient psychological resources to develop and maintain protean career attitudes. According to self-regulation theory, when people pursue goals for achieving personal value, they increase happiness, while pursuing goals for external reasons (for example, because of the attentions from others) leads to dissatisfaction (Sheldon and Elliot, 1998; Ryan and Deci, 2000). Individuals with high core self-evaluations are more confident about themselves and their opinions and they will be looking for goals that they really value rather than pursuing goals just because others value them. The goal of realizing individuals’ value plays an important role between core self-evaluations and goals attainment (Judge et al., 2005), which coincides with the conception of self-orientation and values-driven in the protean career attitudes. Meanwhile, existing studies have shown that there is a significant positive correlation between protean career attitudes and career adaptability (Creed et al., 2010; Chan et al., 2015; Stauffer et al., 2018). According to Direnzo et al. (2015), protean career attitudes make individuals to develop more abilities and resources related to careers, and career adaptability is one of them. Such individuals with protean career attitudes tend to have a strong sense of control, thus promoting the development of career adaptability (Duffy, 2010; Li and Cheung, 2019).

Moreover, based on the distal-proximal framework of motivation (Kanfer, 1990), trait variables, as relatively distal variables, will affect people’s behavior by influencing proximal attitude variables. That is, core self-evaluations, as a distal variable, can influence the protean career attitudes, a proximal variable involving the process of self-regulation, thus exerting an effect on students’ career adaptability. Although both core self-evaluations and protean career attitudes are regarded as adaptive readiness in career construction theory (Neureiter and Traut-Mattausch, 2017; Chui et al., 2020), a large number of previous studies on personality and attitude have told us that personality is the antecedent of attitude (Liao et al., 2008; Lipnevich et al., 2016). Compared with protean career attitudes, core self-evaluations as meta-traits are the distal factors affecting career adaptability, and a causal relationship between core self-evaluations and protean career attitudes has also been demonstrated by existing studies (Rodrigues et al., 2019). At the same time, career construction theory also posits that human development is driven by adaptation to a changing environment through self-construction (Savickas and Porfeli, 2012). As individuals with high core self-evaluations are more likely to have protean career attitudes, and individuals with protean career attitudes are more likely to choose self-directed ways to achieve career goals (Waters et al., 2014), and they will express their values through behaviors (Gubler et al., 2014). In this process of self-regulation, they realize self-construction and ultimately improve their career adaptability. Therefore, we propose the following:

*Hypothesis 2*: protean career attitudes will play a mediating role between core self-evaluations and career adaptability.

### Moderating role of meritocratic beliefs

For young people, in addition to the self-directed and values-driven protean career attitudes will have an impact on career choice and development, their beliefs about the determinants of social attainment will also play a crucial role in the development and pursuit of career aspirations (Laurin et al., 2011; Heckhausen and Shane, 2015). Meritocratic beliefs are precisely beliefs in the principle of distribution of social outcomes, in which a person believes that outcomes should be fairly distributed based on effort or ability (Hu et al., 2020). Since such beliefs usually come from the individual’s daily observation and life experience, reflecting the individual’s cognition of the social public environment (Smith and Stone, 1989). Previous studies have shown that meritocratic beliefs can enhance people’s perceived control and imply the possibility of mobility in society (Hu et al., 2020), thus playing an important role in self-regulation and goal achievement (Bandura and Wood, 1989). We speculate that, meritocratic beliefs may influence the effect of protean career attitudes on career adaptability.

On the one hand, according to the motivation theory related to control beliefs, perceived control plays an important role in the individual’s goal selection and pursuit (Weiner, 1985; Lent et al., 1994; Skinner et al., 1998). At a high level of meritocratic beliefs, people with a protean career attitude will believe that success is based on their efforts and feel more sense of control (Hu et al., 2020), thus promoting the development of career adaptability. However, at low levels of meritocratic beliefs, in which case even people with a protean career attitude will give up those in line with their own values and self-directed career goals, because they do not believe that they can obtain the necessary means to achieve the desired goal (Skinner et al., 1998; Heckhausen et al., 2010), thus they lack the motivation to achieve career goals in a protean environment, which leads to low career adaptability (Li and Cheung, 2019).

On the other hand, meritocratic beliefs not only reflect the idea that outcomes are attributable to individual characteristics but also imply the existence of social mobility (Laurin et al., 2011; Goode et al., 2014). With the influence of high-level meritocratic beliefs, young people are accustomed to exploring new opportunities for self-improvement (Herrmann et al., 2015; Ngo and Hui, 2017), so they have a strong motivation to adapt to the changing environment (Hall, 2002). However, with the influence of low-level meritocratic beliefs, even individuals with protean career attitudes find it difficult to believe that they are likely to pursue better self-development due to their low perception of upward mobility in society (Laurin et al., 2011; Li, 2016). Therefore, we propose the following:

*Hypothesis 3*: meritocratic beliefs will play a moderating role between protean career attitudes and career adaptability.

As mentioned above, protean career attitudes will play a mediating role between core self-evaluations and career adaptability (hypothesis 2), meanwhile, meritocratic beliefs will play a moderating role between protean career attitudes and career adaptability (hypothesis 3). The two hypotheses lay the theoretical foundation for us to build a moderated mediation model in which meritocratic beliefs may moderate the indirect effect of core self-evaluations on career adaptability through protean career attitudes. That is, people’s perceived mobility and control are improved with the influence of meritocratic beliefs (Hu et al., 2020), and the positive impact of protean career attitudes on career adaptability is enhanced, which further strengthens the positive and indirect impact of core self-evaluations on career adaptability. Therefore, we propose the following hypothesis:

*Hypothesis 4*: meritocratic beliefs will moderate the indirect effect of core self-evaluations on career adaptability through protean career attitudes.

**Figure 1:**
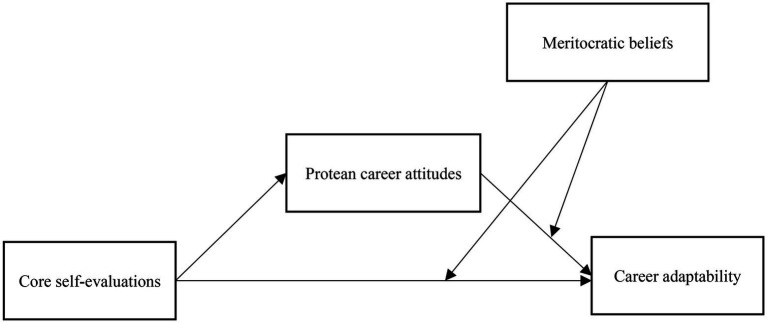
The proposed moderated mediation model.

The conceptual model of the study is presented in Figure 1.

## Materials and methods

### Participants and procedures

A cluster random sampling method was used to select 1,000 students from a college in Chongqing, China. The above variables were measured in turn in 3 weeks. At the first time point (T1), date about personal information was collected, and the core self-evaluations was measured in turn. A week later (T2), protean career attitudes and meritocratic beliefs were measured. Finally, career adaptability was measured in the third week (T3). Besides, this research has set up one item separately: the last four digits of the mobile phone number, so that the data corresponding to the above variables can be effectively matched, and SPSS 23.0 and AMOS17.0 are used to analyze and process the data.

A total of 1,000 complete questionnaires were obtained by matching the last four digits of the mobile phone number, after incomplete questionnaires had been excluded, there were 855(85.50%) valid responses. The characteristics of the sample data are as follows: in terms of gender, 21.5% were men and 78.5% were women; in terms of age, 9.6% are aged 18 to below, 59.9% are aged 19 to 20, 29.4% are aged 21 to 22 and 1.2% are aged over 23; in terms of grades, 45.7% are freshmen, 23.2% sophomores, 28.7% juniors, and 2.5% seniors; in terms of ethnic groups, the Han nationality accounts for 90.6%, and other ethnic minorities account for 9.4%.

### Measures

In this study, we adopted the mature scales to measure the variables. For ensuring the consistency and applicability of the English scale in the Chinese context, the author conducted a translation-back translation procedure (Brislin, 1986). Before the formal investigation, a preliminary test was conducted on 15 college students, and the items were modified according to their feedback.

#### Core self-evaluations

Core self-evaluations were measured with 12 items from the measure developed by Judge et al. (2003) covering four dimensions of self-esteem, self-efficacy, locus of control and neuroticism. A sample items is “I am confident I get the success I deserve in life.” Participants responded on a 5-point Likert scale ranging from 1 (strongly disagree) to 5 (strongly agree). As the factor loading of question 10 of this scale is lower than 0.4, we deleted it. Cronbach’s alpha coefficient of the scale was 0.896, AVE was 0.538, CR was 0.933.

#### Protean career attitudes

Protean career attitudes were measured with a 14-items scale developed by Briscoe et al. (2006), which includes two dimensions: self-directed (e.g., “I am in charge of my own career”) and values-driven (e.g., “It does not matter much to me how other people evaluate the choices I make in my career”). Participants responded on a 5-point Likert scale ranging from 1 (to little or no extent) to 5 (to a great extent). Since the factor loadings of the fourth and fifth questions in the scale were less than 0.4, we deleted these two questions in the subsequent analysis. Cronbach’s alpha coefficient of the scale was 0.910, AVE was 0.521, CR was 0.915.

#### Career adaptability

We used a short version of the Chinese career adaptability scale (Hou et al., 2012). The scale consists of 24 items and measures four career adaptability facets: concern (e.g., “Thinking about what my future will be like”), control (e.g., “Taking responsibility for my actions”), curiosity (e.g., “Exploring my surroundings”), and confidence (e.g., “Taking care to do things well”). Participants were asked to rate how strong their abilities are from 1 (not strong) to 5 (strongest). The overall Cronbach’s alpha coefficient of the scale was 0.971, AVE was 0.570, CR was 0.963.

When some previous studies adopted Hou et al. (2012)‘s scale, they included career adaptability as a global construct into the analysis (Cai et al., 2015; Guan et al., 2015), so our study also referred to their practice.

#### Meritocratic beliefs

Meritocratic beliefs were measured using the 3-item scale devised by Smith and Stone (1989) and modified by Shane and Heckhausen (2015). We have modified the items to make them more relevant to the Chinese context (e.g., “People at the top of the social status ladder in America are there because they have the talent and the ability to succeed” was changed to “People at the top of the social status ladder in China are there because they have the talent and the ability to succeed”). Participants responded on a 6-point Likert scale ranging from 1 (strongly disagree) to 6 (strongly agree). Cronbach’s alpha for this scale was 0.933, AVE was 0.826, CR was 0.935.

#### Control variables

Based on the existing research on the antecedents of career adaptability, demographic variables including gender, age, grade, nationality and major were used as control variables (Hou et al., 2012; Rudolph et al., 2017a,b; Guan et al., 2018). In addition, participation in the internship and earlier participation in extracurricular activities (ECAs) are also considered to have an impact on students’ career adaptability (Monteiro and Almeida, 2015; Ocampo et al., 2020), so they are also included in the control variables.

## Results

### Common method deviation test, reliability, and validity

In this study, Harman’s single-factor was used to test the common method bias since we assessed core self-evaluations, protean career attitudes, meritocratic beliefs and career adaptability using self-report measures. The analysis results showed that the variance explanation rate of the first factor was 28.152%, which did not exceed 40%, indicating that there was no problematic common method bias (Podsakoff and Organ, 1986).

At the same time, we constructed a measurement model including independent variables, mediator variables and dependent variables. And it turned out (see Table 1), the results show that the fitting degree was acceptable compared with other models (χ2/DF = 3.352, CFI = 0.919, TLI = 0.913, GFI = 0.831, RMSEA = 0.052, and SRMR = 0.074), the standardized factor load (λ) of each construct was greater than 0.500, Cronbach’s α and composite reliability (CR) were greater than 0.700. On the square root index of AVE in italics and bold (see Table 2), the values of the involved constructs are all greater than their correlation coefficients with other constructs. This indicates that the construct has good reliability and validity.

**Table 1:** Results of confirmatory factor analysis.

Model	χ^2^/Df	CFI	TLI	RMSEA	SRMR
Benchmark Model: CSE, PCA, CAAS, ME	3.352	0.919	0.913	0.052	0.074
One-Factor Model: CSE, PCA, CAAS_1_, CAAS_2_, CAAS_3_, CAAS_4_, ME	4.068	0.893	0.886	0.060	0.469
Two-Factor Model1: CSE + PCA + CAAS_1_ + CAAS_2_ + CAAS_3_ + CAAS_4_, ME	4.644	0.871	0.865	0.065	0.302
Two-Factor Model2: CSE + ME +CAAS_1_ + CAAS_2_ + CAAS_3_ + CAAS_4_, PCA	4.594	0.873	0.867	0.065	0.301
Three-Factor Model1: CSE + PCA, CA, ME	6.279	0.813	0.804	0.079	0.212
Three-Factor Model2: CSE + PCA + CAAS_1_ + CAAS_2_, CAAS_3_ + CAAS_4_, ME	8.942	0.718	0.705	0.096	0.762
Four-Factor Model1: CSE + ME, PCA + CAAS_1_, CAAS_2_, CAAS_3_ + CAAS_4_	8.432	0.737	0.724	0.093	0.089
Four-Factor Model2: CSE+ CAAS_1_, PCA + CAAS_2_, CAAS_3_ + ME, CAAS_4_	10.589	0.660	0.644	0.106	0.101

CSEs, Core self-evaluations; PCAs, Protean career attitude; ME, Meritocratic beliefs. CAAS, Career adaptability. CAAS_1_ indicates Concern, CAAS_2_ indicates Control, CAAS_3_ indicates Curiosity, CAAS_4_ indicates Confidence.

**Table 2:** Descriptive statistics and correlations of study variables.

	1	2	3	4
1.Core self-evaluations	**0.734**			
2.Protean career attitudes	0.448^**^	**0.722**		
3.Career adaptability	0.454^**^	0.557^**^	**0.755**	
4.Meritocratic beliefs	0.259^**^	0.351^**^	0.311^**^	**0.909**
Mean	3.718	3.647	3.922	4.981
SD	0.573	0.533	0.581	0.879

*N* = 855.

** *p* < 0.01.The bold values represent the square roots of the average variance extracted (AVE) value for each variable.

### Descriptive statistics

Table 1 presents the mean, standard deviation, and correlation coefficients for the study variables. Core self-evaluations correlated moderately with protean career attitudes (*r* = 0.448, *p* < 0.01) and career adaptability (*r* = 0.454, *p* < 0.01), and slightly with meritocratic beliefs (*r* = 0.259, *p* < 0.01). Protean career attitudes correlated moderately with meritocratic beliefs (*r* = 0.351, *p* < 0.01) and career adaptability (*r* = 0.557, *p* < 0.01). Besides, meritocratic beliefs correlated moderately with career adaptability (*r* = 0.311, *p* < 0.01). Although there was only a slight correlation between meritocratic beliefs and core self-evaluations, the correlation between them was significant, which may be related to our large sample size (effective sample size of 855 people), so this result meets the requirements for regression analysis. Meanwhile, all correlation coefficients are below 0.700, indicating that there is no multicollinearity in the data.

### Examining the mediation model

Baron and Kenny’s (1986) causal steps approach is generally used to test mediating effects, However, due to the unreasonable test procedure and the lack of effectiveness of the test method, in recent years, scholars began to recommend the bootstrapping method to directly test the significance of the product coefficient. This paper uses the SPSS macro program process developed by Hayes (2015) to test the mediating role of protean career attitudes in the influence of core self-evaluations on career adaptability.

**Figure 2:**
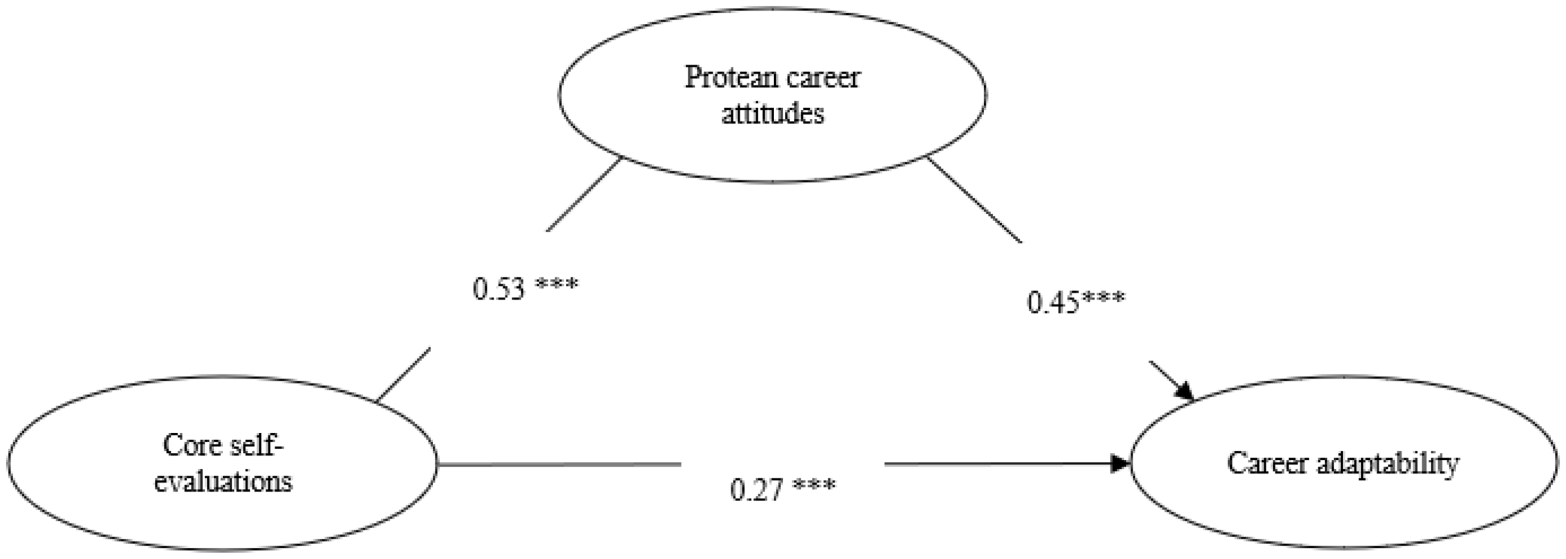
Standardized estimates for the hypothesized mediation model. ***p<0.001.

In Table 3 below, bootstrapping test with 5,000 samples was further used in this paper. It was found that after adding control variables, the total effect of core self-evaluations on career adaptability was 0.454, the 95% confidence interval was [0.457, 0.594], does not include zero, it indicates that core self-evaluations have a significant positive impact on career adaptability, and hypothesis H1 is verified. The indirect effect was 0.218, the 95% confidence interval of the mediating effect was [0.171, 0.269], does not excluding 0. It indicates that protean career attitudes play a mediating role in the effect of core self-evaluations on career adaptability. So, hypothesis H2 is supported. The direct effect was 0.307, the 95% confidence interval of the mediating effect was [0.267, 0.378], does not excluding 0, indicating that after considering the role of protean career attitudes, the effect of core self-evaluations on career adaptability was still significant, indicating that protean career attitudes played a partial mediating role. At the same time, the results of structural equation model verified by AMOS.17 show that hypothesis 1 and Hypothesis 2 still pass the test (see Figure 2).

**Table 3:** Bootstrapping test results for mediating effects.

	Effect	Se	Boot 95% CI	
			LLCI	ULCI
Total effect	0.525	0.035	0.457	0.594
Direct effect	0.307	0.036	0.267	0.378
Indirect effect	0.218	0.025	0.171	0.269

### Testing the moderated mediation model

We also use the SPSS macro program process developed by Hayes (2015) to test the mediating role of protean career attitudes in the influence of core self-evaluations on career adaptability. In order to avoid multicollinearity, all interaction terms are centralized before hierarchical regression, and the regression results of the moderating effect are shown in Table 4. The interaction between protean career attitudes and meritocratic beliefs had a significant positive effect on career adaptability (*β* = 0.148, *p* < 0.001), H3 is supported.

**Table 4:** Bootstrapping test results of the moderation effect.

Variables	Coeff	se	t	*P*	LLCI	ULCI
Constant	3.819	0.117	32.712	0.000	3.590	4.048
Protean career attitudes	0.557	0.034	16.246	0.000	0.490	0.625
Meritocratic beliefs	0.110	0.020	5.525	0.000	0.071	0.149
Protean career attitudes * meritocratic beliefs	0.148	0.033	4.514	0.000	0.084	0.212
Gender	0.010	0.040	0.242	0.809	−0.069	0.088
Age	−0.076	0.036	−2.126	0.034	−0.146	−0.006
Grade	0.062	0.025	2.489	0.013	0.013	0.111
Nationality	0.017	0.044	0.392	0.696	−0.069	0.103
Major	0.006	0.007	0.878	0.380	−0.008	0.020
Participation in the internship	−0.014	0.042	−0.326	0.745	−0.096	0.069
Earlier participation in ECAs	0.079	0.041	1.956	0.051	0.000	0.159

The symbol “*” represents the interaction between Protean career attitudes and protean career attitudes.

In order to avoid multicollinearity, all interaction terms are centralized before hierarchical regression, and the regression results of the moderating effect are shown in Table 1. The interaction between protean career attitudes and meritocratic beliefs had a significant positive effect on career adaptability (*β* = 0.061, *p* < 0.001), H3 is supported.

**Figure 3:**
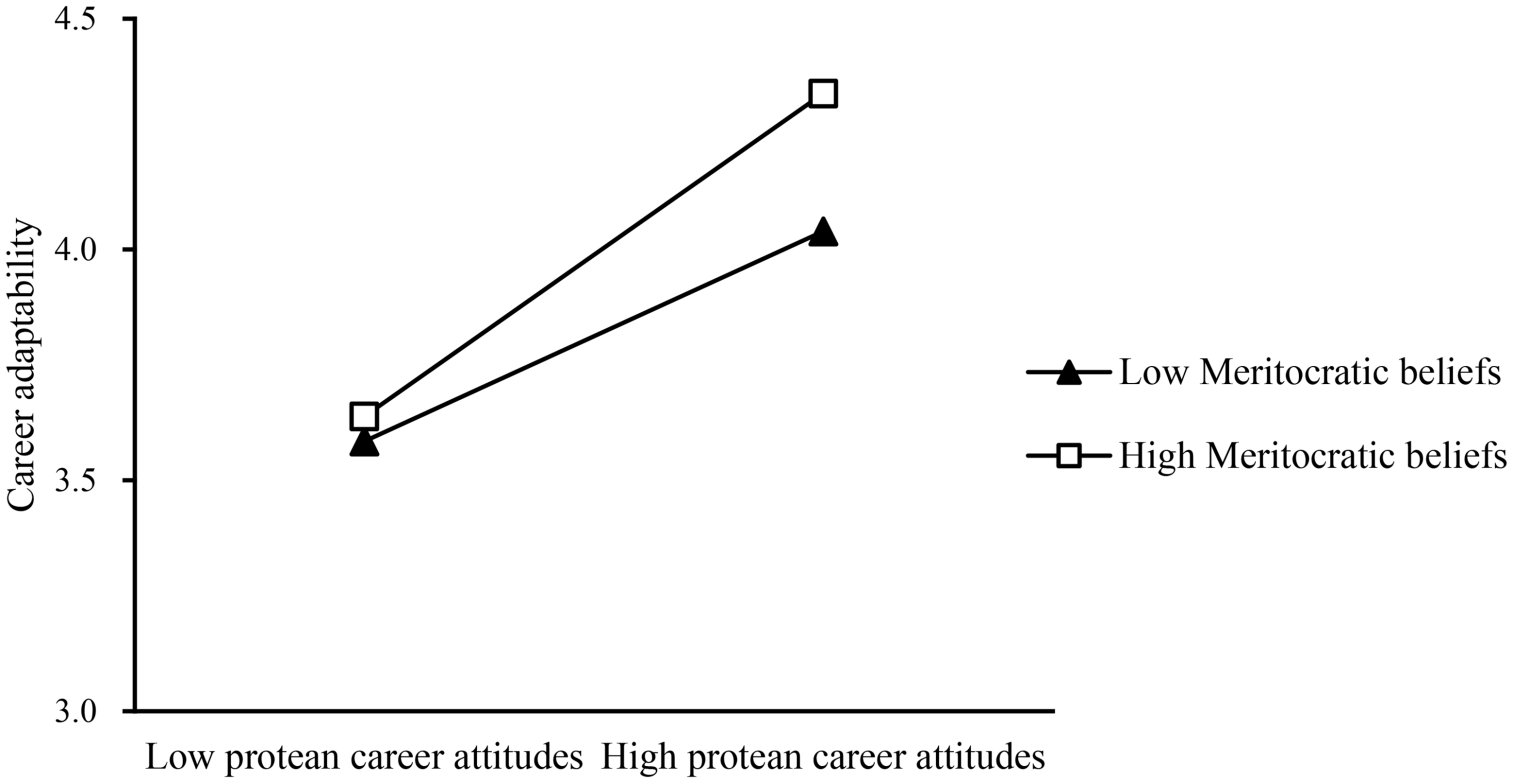
Interactive effect of protean career attitudes and meritocratic beliefs on career adaptability.

To more intuitively show the moderating role of meritocratic beliefs, we plotted the moderating effect figure based on one standard deviation above and one standard deviation below the mean, respectively. As shown in Figure 3, when the meritocratic beliefs are at a high level, the positive effect of protean career attitudes on career adaptability is enhanced, while when the meritocratic beliefs are at a low level, the impact of protean career attitudes on career adaptability is not significant. Therefore, high meritocratic beliefs can strengthen the impact of protean career attitudes on career adaptability.

As shown in Figure 3, when the meritocratic beliefs at higher levels, protean career attitudes play a positive role in enhancing career adaptability, and when the lower level of meritocratic beliefs, protean career attitudes affect career adaptability difference, therefore, high meritocratic beliefs can strengthen the protean career attitudes affect career adaptability, H3 is supported again.

To examine the moderated mediation effect proposed in hypothesis 5, the Bootstrap method was used to test the robustness of the moderated mediating effect of meritocratic beliefs. As shown in Table 5, the 95% confidence interval of the high meritocratic beliefs group was [0.171, 0.271], excluding 0, and the indirect effect value was greater than that of the other groups; the 95% confidence interval of the low meritocratic beliefs group was [0.093, 0.183], excluding 0. Index of moderated mediation following Hayes (2015) is 0.047, 95% confidence interval is [0.025, 0.073], excluding 0. Therefore, H4 is valid.

**Table 5:** Bootstrap test results of the moderating mediation effect.

Meritocratic beliefs	Effect	SE	LLCI	ULCI
−1SD	0.136	0.023	0.093	0.183
Mean	0.178	0.022	0.138	0.222
+1SD	0.219	0.025	0.171	0.271

## Discussion

Career adaptability refers to a psychosocial construct that denotes an individual’s resources for coping with current and anticipated tasks, transitions, traumas in their occupational roles (Savickas and Porfeli, 2012). We take college students as the research object, and establishes a theoretical model of core self-evaluations—protean career attitudes—career adaptability based on career construction theory and self-regulation theory, and examines the moderating role of meritocratic beliefs between protean career attitudes and career adaptability. This study responds to the call from predecessors that future research could explore the mediating mechanisms leading from adaptivity to the development of adaptability and the moderators or the conditions under which adaptivity leads to higher adaptability (Hirschi and Valero, 2015). From the perspective of protean careers, our research not only emphasizes the possibility of individual inner mobility (that is, protean career attitudes), but also pays attention to the existence of external mobility (that is, meritocratic beliefs), thereby further revealing the antecedent mechanism of career adaptability and provides a reference for promoting colleges to cultivate college students’ career adaptability.

### Theoretical implications

Firstly, based on the data analysis results, we verified the positive effect of core self-evaluations on career adaptability (Hypothesis 1) and the mediating effect of protean career attitudes between core self-evaluations and career adaptability (Hypothesis 2). Some previous studies have confirmed the positive correlation between core self-evaluations and career adaptability (Zacher, 2014; Rudolph et al., 2017a,b), and our study has again proved this positive relationship, so we do not need to go into details about it. However, there is a lack of effective discussion on the mediating mechanism between core self-evaluations and career adaptability (Hirschi and Valero, 2015). And the verification of Hypothesis 2 suggests that, in the complex and uncertain environment, understanding the role of protean career attitudes can help us better understand how adaptivity affects the development of adaptability. In addition, although career construction theory (Savickas, 2005; Savickas, 2013) outlines a sequential model from adaptivity to adaptability, adapting, and adaption (Savickas, 2013), no research has pointed out that there is a sequential relationship between adaptive readiness (i.e., adaptivity). According to the proximal and distal framework about motivation (Kanfer, 1990, 1992; Barrick, 2005), distal personality traits can influence individual behavior through proximal constructs (such as motivational states). As personality traits, core self-evaluations can effectively predict attitudes, motivations, and behaviors (Judge and Kammeyer-Mueller, 2011), and individual personality trait is also one of the important antecedents of protean career attitudes (Rodrigues et al., 2019). Therefore, the verification of Hypothesis 2 reminds us that there is also a sequential relationship between adaptivity (i.e., core self-evaluations) and adaptivity (i.e., protean career attitudes). In addition, it is worth noting that according to Table 4, the two factors of age and grade have a significant impact on career adaptability, and previous studies also showed that there are significant differences in career adaptability among students of different ages and grades (Hou et al., 2012; Rudolph et al., 2017a,b), which may be related to the fact that older or older students have accumulated more career experience or are more concerned about employment information. Therefore, in order not to affect the investigation of the main effects, we controlled for their effects during the study.

Second, the verification of hypothesis 3 showed that meritocratic beliefs positively moderated the relationship between protean career attitudes and career adaptability, and the important implication of this finding is that in exploring the antecedents of the development of career adaptability, we realized the combination of self-mobility and social mobility, and the combination of perceived self-control and external-control (i.e., the combination of protean career attitudes and meritocratic beliefs). In other words, in order for young people with protean career attitudes to be truly free to pursue better career development, they need to own the perception of external-control and a belief in the possibility of social mobility. Previous studies have shown that meritocratic beliefs have many positive effects, such as helping adolescents improve their mental health and life satisfaction (Weinberg et al., 2020), providing motivations for individuals to increase their engagement in career goals (Shane and Heckhausen, 2015), and increasing their income (Hu et al., 2020). However, in the western economic and political environment, other studies have shown the negative effects brought by meritocratic beliefs, such as the aggravation of social inequality by justifying prejudice and discrimination through institutional rationalization (Ledgerwood et al., 2011; Mijs and Savage, 2020). However, in the Chinese context, our research again supports the positive effect of meritocratic beliefs, which, as a moderating variable representing college students’ social environment perceptions, are of great significance for college students’ career adaptability. With the influence of meritocratic beliefs, young people can obtain any career they want within the scope of their efforts and abilities (Shane and Heckhausen, 2013). With perceived social mobility and control, they with protean career attitudes are more likely to develop a higher level of career adaptability.

### Practical implications

First, the results show that core self-evaluations have a positive impact on college students’ career adaptability *via* protean career attitudes. Therefore, on the one hand, on the basis of understanding the antecedent of differences in career adaptability, career counselors could attempt to identify the state of adaptive readiness of different student groups through standardized assessments (Judge et al., 2003). Since students with low adaptivity usually exhibit low levels of adaptability (Hirschi et al., 2015), counselors should offer interventions that aim to increase their adaptability or directly facilitate their adaptive attitudes and behaviors (Koen et al., 2012). On the other hand, by further understanding the distal and proximal predictors of career adaptability, this study provides educators and counselors with practical knowledge of adaptation tendencies. The discovery of the mediating role of protean career attitudes can help practitioners design interventions related to specific indicators of low adaptability in order to better engage individuals in self-improvement activities for optimal adaptability (Betz, 2004).

Second, the result highlights the importance of meritocratic beliefs in young people’s career self-regulation. Meritocratic beliefs can positively moderate the effect of protean career attitudes on career adaptability. When college students perceive the opportunity to obtain higher socioeconomic status, they will conduct motivational self-regulation (Shane and Heckhausen, 2015). Our findings suggest that the belief that a person’s social outcomes depend on their personal merit might facilitate their actions related to social mobility. To enable young people to pursue their career goals more freely, career counselors we can help them build meritocratic beliefs in education, for example by rewarding students who do well in the task and making them believe they can develop themselves through hard work (Hu et al., 2020). At the same time, it is a reminder that although protean career attitudes deemphasize traditional hierarchical upward mobility and allow individuals to flow with internal self-direction, we must also pay attention to whether society provides mobility possibilities for students. Meritocratic belief is not only a psychological perception, but also reflects the social public environment to some extent because it is usually derived from daily observations, experiences, and lay philosophies (Smith and Stone, 1989), therefore, we call for the society to provide more mobility possibilities for young people in the future.

### Limitations and future directions

There are still some limitations in our study, which can be improved in future studies.

First, career construction theory also indicates that there may be a dynamic correlation between individual adaptive resources (such as core self-evaluations and protean career attitudes) and career adaptability (Savickas, 2005; Savickas and Porfeli, 2012), high level core self-evaluations promotes the development of college students’ career adaptability through protean career attitudes, while the improvement of career adaptability also brings positive results, such as career success (Haenggli and Hirschi, 2020), which in turn improves individual self-evaluations. Therefore, we suggest exploring this dynamic mechanism in future studies.

Second, we may be able to explore more personal or situational boundary mechanisms to enrich our research, such as subjective socioeconomic status, which represents the outcome of social distribution and is closely associated with meritocratic beliefs (Shane and Heckhausen, 2015; Weinberg et al., 2020). We can measure the gap between beliefs and reality through a 2 (high meritocratic beliefs and low meritocratic beliefs) × 2 (high and low subjective socioeconomic status) experimental between subjects’ factorial design, and explore the interaction between the two factors to make the study more realistic.

Finally, although we measured the variables involved in the study at three time points, we did not measure all variables at each time point, which may amplify the causality between variables. Therefore, we suggest that longitudinal follow-up investigation can be used to delve into this topic in the future, to draw more convincing evidence.

## Data availability statement

The raw data supporting the conclusions of this article will be made available by the authors, without undue reservation.

## Ethics statement

Ethical review and approval was not required for the study on human participants in accordance with the local legislation and institutional requirements. The patients/participants provided their written informed consent to participate in this study.

## Author contributions

NL wrote the manuscript and analyzed the data under the guidance of BD and XY. XL contributed to data analysis and editing of the manuscript. BD contributed to study design and data collection. XY contributed to study design and critical revisions. All authors contributed to the article and approved the submitted version.

## Funding

This work was supported by Luzhou Key Research Base of Philosophy and Social Sciences · Luzhou Social Public Security Research Center project: Research on the path of urban public safety management and resilience governance (SHAQ202209), 2022 Annual project of Sichuan Party History and Party Construction Research Center: Research on the Application of “Lucky Party Building” Group Guidance in University Students’ Party Building Work (DSDJ22-14), 2022 Annual Project of Regional Public Management Informatization Research Center: Research on the path of new cloud computing service System supporting Sichuan Digital Village Construction (QGXH22-05), and Chengdu social Science Planning project: The impact of industrial metaverse on the development of digital industry in Chengdu and risk prevention and control research (2022CS053).

## Conflict of interest

The authors declare that the research was conducted in the absence of any commercial or financial relationships that could be construed as a potential conflict of interest.

## Publisher’s note

All claims expressed in this article are solely those of the authors and do not necessarily represent those of their affiliated organizations, or those of the publisher, the editors and the reviewers. Any product that may be evaluated in this article, or claim that may be made by its manufacturer, is not guaranteed or endorsed by the publisher.
